# Multi-Task and Federated Learning for Breast and Lung Cancer Screening and Diagnosis: A Survey and Future Research Directions

**DOI:** 10.3390/jimaging12060258

**Published:** 2026-06-11

**Authors:** Alexandru Ciobotaru, Cosmina Corches, Dan Gota, Liviu Miclea

**Affiliations:** Automation Department, Faculty of Automation and Computer Science, Technical University of Cluj-Napoca, 400114 Cluj-Napoca, Romania; alexandru.ciobotaru@aut.utcluj.ro (A.C.); cosmina.corches@aut.utcluj.ro (C.C.); dan.gota@aut.utcluj.ro (D.G.)

**Keywords:** breast cancer, lung cancer, multi-task learning, deep learning, federated learning, medical image analysis

## Abstract

Background: Breast cancer (BrC) and lung cancer (LuC) are two forms of aggressive cancer that affect both men and women worldwide. Recently, multitask learning (MTL) and federated learning (FL) techniques have proven to be efficient in increasing the robustness of deep learning (DL)-based models by performing multiple tasks simultaneously and preserving the confidentiality of medical data. Methods: This paper presents a survey of MTL and FL methods for BrC and LuC screening and diagnosis using the Preferred Reporting Items for Systematic Reviews and Meta-Analyses (PRISMA) methodology. Comprehensive tables were created to highlight the performances of both MTL models and FL environments. Results: The main challenges identified were the lack of hybrid MTL models that combine hard and soft sharing, heterogeneous imaging data, and edge FL systems. Conclusions: FL environments obtain competitive performance compared with centralized MTL models, highlighting their potential to preserve medical data confidentiality without compromising performance. Future research directions could include MTL-based models incorporated in FL environments, hybrid MTL models that combine both hard- and soft-sharing parameter methods, and the use of blockchain techniques to increase the security of FL environments.

## 1. Introduction

**Figure 1:**
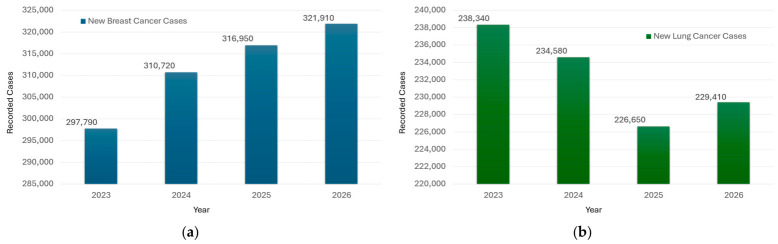
Overview of the trend regarding the newly recorded (**a**) BrC and (**b**) LuC cases between 2023 and 2026 according to American Cancer Society.

Breast Cancer (BrC) is one of the most lethal diseases that affect women worldwide. According to the American Cancer Society, 321,910 new BrC cases were estimated solely in the USA by 2026 [1]. As shown in Figure 1a, the number of newly recorded female BrC cases has been constantly increasing during the post-pandemic period: 297,790 in 2025 [2], 310,720 in 2024 [3], and 316,950 in 2023 [4].

Lung Cancer (LuC) is another major disease that affects both men and women. Therefore, the number of newly recorded LuC cases in 2026 was 229,410. As shown in Figure 1b, the number of newly recorded LuC cases for both male and female in the post-pandemic period follows a relatively decreasing trend: 226,650 in 2025 [2], 234,580 in 2024 [3] and 238,340 in 2023 [4]. However, the number of LuC cases began to increase in 2026 compared with the previous year.

In recent years, automated deep learning (DL)-based computer aided diagnosis (CAD) systems specifically designed for BrC and LuC screening and diagnosis have shown remarkable results in the classification and detection of breast lesions [5] and lung tumors [6]. Typical DL-based tasks that are performed on medical images include classification, segmentation, and detection. While a significant number of papers in this field have focused on a single task (i.e., mostly classification and segmentation), recently, multi-task learning (MTL)-based models have been proposed. Such models can focus on at least two tasks simultaneously (i.e., lesion classification and segmentation), thus contributing to increased diagnostic reliability and assisting radiologists in diagnosing the advancement of cancer based on the BiRADS level.

Another major issue related to DL-based cancer screening and diagnosis is the limited publicly available data. Most publicly available datasets are limited to at least a few thousand images, which negatively affects the generalizability of models. Therefore, collection of high-quality medical images is usually time-consuming. Moreover, the cost of creating a large and secure server that can host these images may be relatively high.

Most current systematic literature reviews have focused on the performance of unimodal single-task DL-lung [6] or breast cancer [7]. The main contributions of this paper are as follows:

- A comprehensive overview of recent centralized MTL and FL as emerging methods highlights their roles in enhancing tumor detection efficiency and medical data privacy for BrC and LuC screening and diagnosis.
- A comparative performance analysis of the MTL models and FL environments in terms of accuracy, recall, and F1-score was visualized through box plots.
- An outline of current challenges and future directions in the context of MTL and FL methods for BrC and LuC screening and diagnosis.

The remainder of this paper is organized as follows. Section 2 presents the methodology used to select and filter included studies. Section 3 describes the formulation of MTL and FL paradigms. Section 4 presents existing work on MTL and FL for BrC screening and diagnosis. Section 5 presents work on MTL and FL for LuC screening and diagnosis. Section 6 compares the performance reported in the studies presented in Section 4 and Section 5 and highlights several challenges and future research directions. Finally, Section 7 concludes the study.

## 2. Review Methodology

**Figure 2:**
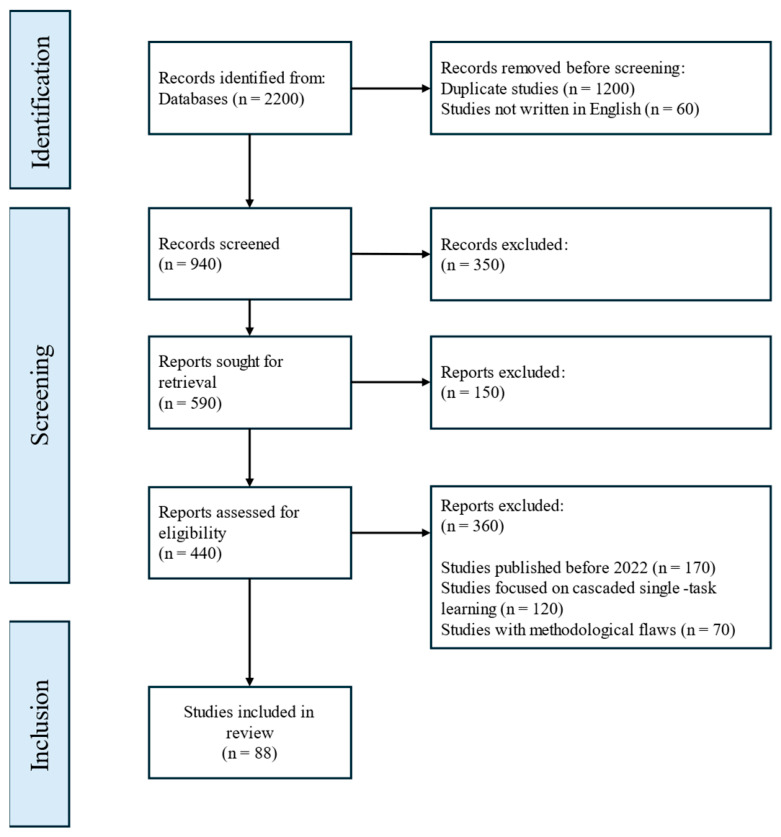
Flow diagram of PRISMA methodology.

The review methodology used to select and filter papers is represented by Preferred Reporting Items for Systematic Reviews and Meta-Analyses (PRISMA), as shown in Figure 2. Several scientific databases were utilized to select relevant papers: Clarivate Web of Science, Scopus, PubMed, and Google Scholar search engines. The search queries included the following principal terms: “deep learning”, “multi-task learning”, “federated learning”, “breast cancer” and “lung cancer”. To provide a better overview of the initial screening process, the search queries are listed below.

- [Breast Cancer OR Lung Cancer] AND Multi-Task Learning AND [Ultrasound Images OR Mammography OR Histopathological Images OR Fine Needle Aspirate OR Computed Tomography].
- [Breast Cancer OR Lung Cancer] AND Multi-Task Learning AND Convolutional Neural Network AND CNN AND [Ultrasound Images OR Mammography OR Histopathological Images OR Fine Needle Aspirate OR Computed Tomography].
- [Breast Cancer OR Lung Cancer] AND Multi-Task Learning AND Vision Transformer AND ViT AND [Ultrasound Images OR Mammography OR Histopathological Images OR Fine Needle Aspirate OR Computed Tomography].
- [Breast Cancer OR Lung Cancer] AND Federated Learning AND FL AND [Ultrasound Images OR Mammography OR Histopathological Images OR Fine Needle Aspirate OR Computed Tomography].

The inclusion and exclusion criteria used in the filtered studies are presented in Table 1. First, only articles that were written in English were included. Second, we selected studies that focused on both MTL- and FL-based methods using not only traditional CNN architectures (i.e., ResNet, XceptionNet, etc.) but also hybrid CNN models that integrate attention-based modules and pure ViT baselines. Additionally, only articles published between 2022 and 2026 that focused on MTL and FL methods for BrC and LuC screening and diagnosis were considered to capture the most recent advancements in this field.

**Table 1:** Inclusion and Exclusion Criteria.

No.	Inclusion Criteria	Exclusion Criteria
1.	Articles published in English	Articles published in other languages than English
2.	Articles that utilize in the context of centralized MTL both traditional CNN models as well as hybrid attention-enhanced CNN architectures and ViT models for BrC and LuC screening and diagnosis	Articles covering centralized unimodal methods for BrC and LuC screening and diagnosis
3.	Articles that propose decentralized FL environments for BrC and LuC screening and diagnosis	Articles with methodological flaws or with incomplete results presentation.
4.	Articles from the 2022–2026 time frame	Articles published before 2022

On the other hand, articles that are focused on centralized unimodal DL-based methods for BrC and LuC screening and diagnosis as well as papers with methodological flaws or incomplete results reports were not included in the survey.

**Figure 3:**
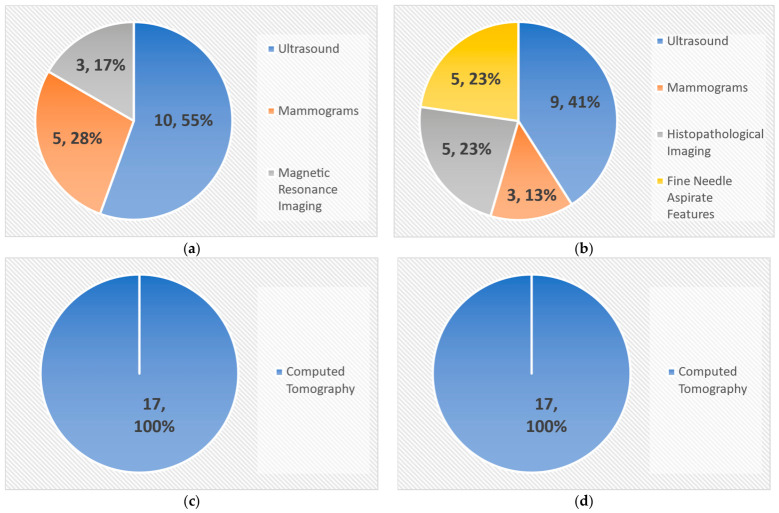
Distribution of the number of studies and corresponding percentages according to the imaging modality used in (**a**) MTL-based BrC analysis, (**b**) FL-based BrC analysis, (**c**) MTL-based LuC analysis, and (**d**) FL-based LuC analysis.

Figure 3 shows the distribution of studies addressing MTL- and FL-based screening and diagnosis of BrC and LuC, together with the datasets described in these studies, allowing a clearer comparison of the approaches and data considered in a continuously evolving research field. The labels indicate the number of studies and their corresponding percentage. The main imaging techniques used for BrC screening and diagnosis are ultrasonography, mammography, magnetic resonance imaging, histopathology and, fine needle aspirate features extracted from digitalized images. On the other hand, the imaging techniques used for LuC screening and diagnosis include computed tomography (CT).

## 3. Formulation of Multi-Task and Federated Learning

### 3.1. Multi-Task Learning

**Figure 4:**
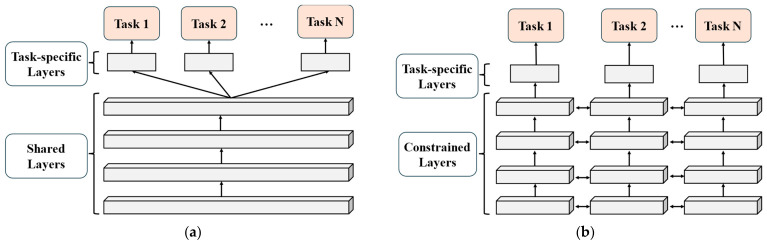
(**a**) Hard and (**b**) soft parameter sharing methods in MTL for medical image analysis.

Single-task DL-based methods achieve notable results for both BrC and LuC medical image analysis by minimizing a single loss function corresponding to a DL architecture (i.e., typically a CNN or vision transformer (ViT) model). However, by employing MTL, the generalization and robustness of DL-based CAD systems are increased by forcing DL models to learn a shared representation that captures the underlying patterns common to multiple tasks, thereby acting as a type of inductive transfer [8]. In addition, inductive bias tends to cause an architecture to favor certain hypotheses over others, thereby reducing overfitting. Therefore, MTL is particularly beneficial in medical image analysis for breast and lung cancer, where high-quality labeled data are scarce and time-consuming. From a DL perspective, MTL can be classified into two types: hard and soft parameter sharing of hidden layers, as shown in Figure 4.

Hard parameter sharing (Figure 4a) is an MTL-based method in which multiple shared hidden layers common to all tasks are exposed and utilized for feature extraction from medical images. Next, several output task-specific layers are maintained to further process the information for each task. Formally, given T tasks and a shared encoder $h=f_{\theta }(x)$ characterized by the common feature extractor function $f_{\theta }(x)$, the task-specific heads can be expressed according to Equation (1).

(1) $$\hat{y^{T}}=g_{\phi_{T}}\left(h\right)=g_{\phi_{T}}\left(f_{\theta }(x)\right)$$

where $\phi_{T}$ represents task-specific trainable parameters. For each task T, the task-specific loss function can be expressed using Equation (2).

(2) $$L_{T}(\theta ,\;\phi_{T})=\frac{1}{N_{T}}\sum_{i=1}^{N_{T}}l(g_{\phi_{T}}\left(f_{\theta }\left(x_{i}^{t}\right)\right),\;y_{i}^{t})$$

where $N_{T}$ represents the number of training observations belonging to task T. Depending on the nature of the specific task, different types of loss functions are utilized (i.e., cross-entropy function for multi-label classification tasks, binary cross-entropy function for binary classification tasks, or dice loss for segmentation tasks). Finally, the overall MTL loss can be computed as a joint optimization objective function according to Equation (3).

(3) $$L_{hard-sharing}=\sum_{i=1}^{T}\lambda_{i}\cdot L_{T}(\theta ,\;\phi_{T})$$

The advantage of the hard parameter-sharing method lies in its ability to reduce overfitting compared to single-task learning. In fact, the more parallel tasks that are employed, the fewer the chances there are for the model to overfit. However, the tasks are very different and the performance of the DL-based architecture can be negatively affected.

In the soft parameter-sharing (Figure 4b) paradigm, a specific DL-based model is assigned to each task. In addition, the distance between the models was regularized using a specific function $\Omega (\theta_{T})$ to minimize it. Thus, the joint objective function can be expressed using Equation (4), where α controls the level of coupling between the models for each task T.

(4) $$L_{soft-sharing}=\sum_{i=1}^{T}\lambda_{i}\cdot L_{T}\left(\theta ,\;\phi_{T}\right)+\alpha \cdot \Omega (\theta_{T})$$

### 3.2. Federated Learning

**Figure 5:**
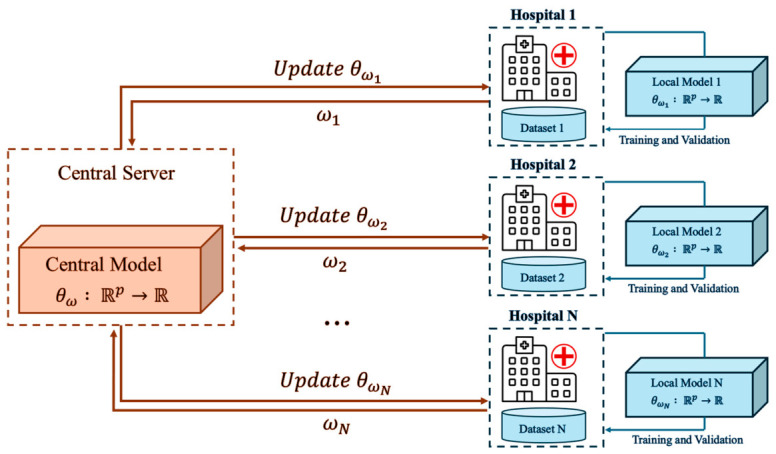
Overview of a Federated Learning environment composed of n local clients and a central server for breast and lung cancer analysis using medical imaging.

Federated learning (FL) has emerged as a reliable and efficient method of decentralized training manner with multiple clients (i.e., hospitals or medical institutions), as depicted in Figure 5. Each client uses its own data to train a local DL-based model, and aggregates only the trainable parameters (i.e., weights) on a central DL-based model, which is usually located on a server. Thus, the data confidentiality at the client-side level was preserved. In addition, the scalability of the federated architecture is higher than that of the centralized approach because newer clients can be easily integrated into the environment.

**Figure 6:**
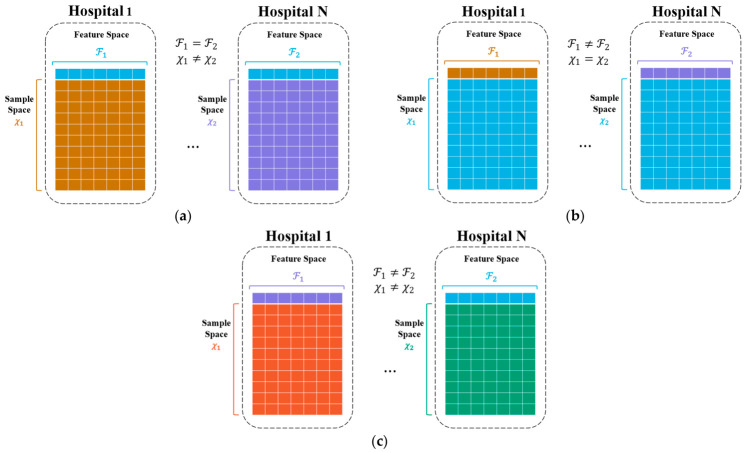
Overview of (**a**) Horizontal, (**b**) Vertical and (**c**) Transfer FL Environments.

From a data distribution perspective, FL environments can be categorized into three types: horizontal, vertical, and transfer-learning [9], as shown in Figure 6:

- Horizontal FL Environments: The feature space $F_{k}$ is the same across the client side but the sample space $\chi_{k}$ is different. In this case, all hospitals within the FL environment contained the same medical images for all patients (i.e., CT scans, mammograms, ultrasound images, etc.). However, each hospital had different patients and implicit types of medical images (i.e., samples).
- Vertical FL Environments: The feature space is different for each client, but the sample space is the same. In this case, the same group is considered for every medical institution; however, each institution has different medical information (e.g., CT scans, ultrasound images, genomic markers, and biopsy reports).
- Transfer Learning FL Environments: The feature and sample spaces are different for each client. Therefore, this scenario best models the real-life situation of a federated environment, in which different hospitals have different patients, each with various types of medical information related to BrCs and LuCs.

Table 2 summarizes the characteristics of each FL environment type along with the identified advantages and associated challenges.

**Table 2:** FL environment types based on data distribution.

Type	Identical Feature Space	Identical Sample Space	Advantages	Challenges
Horizontal	✓	✗	Simple and clear aggregation methods.	Non-identically distributed configurations.
			Suitable for multi-task learning methods.	May become unrealistic in real-life federated scenarios.
			Easily scalable.	Easy to implement and maintain.
Vertical	✗	✓	Suitable for multi-modal learning methods.	Domain-shift in terms of sample space between clients.
			Suitable for modeling departments within the same hospital.	Only overlapping samples can participate in the environment.
				Harder to implement and increase maintenance costs.
Federated Transfer Learning	✗	✗	Most realistic federated configuration.	Domain-shift in terms of both features and sample space between clients.
			Increased per-client confidentiality.	Harder to implement and increase maintenance costs.
			Flexible in terms of client data type.	

Although FL increases data confidentiality by enabling locally distributed learning, the information between local clients and the central server can be intercepted and altered by external cyber-attacks. Therefore, additional security methods should be integrated into FL environments to further strengthen medical-image security. Thus, based on the surveyed papers, we further categorized FL environments based on whether additional encryption techniques have been integrated:

- Pure FL Environments: Environments in which data confidentiality is maintained based solely on the decentralized configuration ensured by the federated paradigm.
- Encryption-enhanced FL Environments: Environments in which data confidentiality is increased by utilizing encryption methods (e.g., homomorphic encryption) on either the client or server sides. In addition, environments that utilize DL-based methods to combat adversarial attacks are considered in this category.

The reviewed studies primarily utilized two types of aggregation techniques: federated average (FedAvg) and FedProx. Given K clients, $s_{k}$ samples per client, and N samples, the weights $\theta_{t+1}$ at round t + 1 can be computed according to Equation (5). Despite its simplicity and computational efficiency, the limitations of this aggregation technique include relatively high client drifts in non-identically distributed data and heterogeneous system scenarios.

(5) $$\theta_{t+1}=\frac{1}{N}\sum_{i=1}^{K}s_{k}\theta_{t}^{K}$$

In contrast, the FedProx objective [10] imposes a proximal term, usually denoted by μ in order to limit the client drift from the global model. Therefore, for a client within the FL environment, the FedProx objective can be computed using Equation (6).

(6) $$\theta_{t+1}=\underset{\theta }{\mathrm{argmin}}\left(L\left(\theta \right)+\frac{\mu }{2}{\left\|\theta -\theta_{t}\right\|}^{2}\right)$$

where $L\left(\theta \right)$ denotes the loss function of the local client. If the value of μ is zero, no regularization is applied to the local models. Similarly, if the value of μ is large, local models will not be able to generalize the information received from the global model. Therefore, for heterogeneous data, the optimal value interval for the proximal coefficient μ was between 0.1 and 1.

From a theoretical point of view, FedAvg and FedProx are efficient; however, in real clinical environments, they have several limitations. For instance, in real-world non-identically distributed (non-IID) contexts with severe data heterogeneity, FedAvg may suffer from client drift, because it assumes similar local gradients. While FedProx lightly addresses this limitation, large proximal coefficient values degrade the robustness of the local DL models. Recent studies have addressed these limitations by proposing personalized FL environments. For example, the study conducted by Sun et al. et al. [11] used pHash-derived Hamming distances to create a client similarity matrix, and proposed a hypernetwork that generates personalized client-specific parameters. Thus, the inter-client data distribution discrepancy is attenuated, thereby enhancing the prediction robustness. Paper [12] proposed a personalized FL environment that exchanges class prototypes instead of using a full model weight. Therefore, the conflicting gradient problem is avoided because the model weights of the individual clients are not shared with the server. Moreover, Niu et al. [13] proposed FedCGP, which incorporates a two-stage aggregation technique instead of a single uniform averaging method to address client-side discrepancies. After grouping clients with similar data distributions, a homogenous aggregation method (e.g., FedAvg) was employed to extract common features. In the second stage, personalized aggregation starts once a cluster converges and is verified by gradient norm conditions.

## 4. Multi-Task and Federated Learning for Breast Cancer

### 4.1. Multi-Task Learning Methods for Breast Cancer Screening and Diagnosis

Table 3 presents the reviewed papers covering MTL methods for BrC screening and diagnosis. For ultrasound-based diagnosis, BUSI [14], UDIAT [15], and OASBUD [16] datasets were employed. CBIS-DDSM [17] and InBreast [18] have been employed for BrC diagnosis using mammograms. First, several reviewed papers validated the models on multiple datasets [19,20,21,22], enforcing their reliable performance on different imaging sources. Notably, most of the reviewed studies implemented hard-sharing MTL methods in which a common feature extractor was employed for feature extraction, which is also enriched with attention mechanisms [20,23]. However, in a recent study conducted by Sun et al. [24], a hybrid approach between hard and soft parameter sharing was proposed by utilizing the shared low-level information across both tasks (i.e., histological grade and Ki-67 status) while preserving task-specific discriminative information learned at a higher semantic level. From a clinical perspective, the interpretability of DL-based models is a critical factor, because it highlights the reasoning behind these architectures, thus being a useful debugging tool. Additionally, they can assist junior radiologists in the screening process. In the context of medical image analysis, one of the most widely utilized explainable AI (XAI) methods are represented by Grad-CAM diagrams. They highlighted the areas that were most influential in the decision-making process of DL models. Therefore, several studies use Grad-CAM diagrams, in the context of MTL-based BrC screening and diagnosis [20,22,23,24,25].

**Table 3:** Summary on MTL Methods for BrC Screening and Diagnosis.

Ref.	Tasks	Parameter Sharing	Imaging Technique	Dataset	DL Architecture	Performance [%]
[26]	Classification + Segmentation	Hard Sharing	Ultrasound	BUSI	nnU-Net	Acc: 84.8 Recall: 79.0 F1-Score: 84.6 Dice: 79.3
					UNet ++	Acc: 85.8 Recall: 80.5 F1-Score: 85.8 Dice: 80.3
[19]	Density Classification + Mass Segmentation	Hard Sharing	Mammograms	CBIS-DDSM	Res2Net101 + ViT Encoder–Decoder	Acc: 86.0 Recall: 86.0 F1-Score: 87.98 Dice: 89.8
				INBreast		Acc: 96 Recall: 99 F1-Score: 97.5 Dice: 91
[20]	Joint Classification + Segmentation	Hard Sharing	Ultrasound	UDIAT	Res-U-Net + OCA module	Acc: 94.79 Recall: 95.35 F1-Score: 94.9 Dice: 84.85
				OASBUD		Acc: 91.67 Recall: 90.13 F1-Score: 91.63 Dice: 83.75
[23]	Segmentation + pCR prediction	Hard Sharing	Magnetic Resonance Imaging	TCIA Duke Dataset	3D Attention UNet model + MLP	Acc: 76.7 Recall: 78.0 F1-Score: N/A Dice: 76.9
[25]	Segmentation + Biomarker Prediction	Hard Sharing	Ultrasound	3D Whole Ultrasound Images	3D ResNet encoder–decoder + fully connected network	Acc: 58.8 Recall: 69.4 F1-Score: 73.8
[24]	Histological Grade Prediction	Hybrid Sharing	Magnetic Resonance Imaging	Private Dataset collected from 301 patients	DenseNet + task common and task specific network	Acc: 87.1 Recall: 87.2 F1-Score: 87.6
	Ki-67 status Forecasting					Acc: 77.7 Recall: 95.3 F1-Score: 84.6
[21]	Classification + Segmentation	Hard Sharing	Ultrasound	BUSI	UNet + Gated Unit Modules	Acc: 94.44 Recall: 93.86 F1-Score: 94.23 Dice: 84.9
				UDIAT		Acc: 88.96 Recall: 87.52 F1-Score: 88.39 Dice: 89.12
[27]	Pathology Prediction	Hard Sharing	Mammograms	INbreast	EfficientNet-B3 + Attention Mechanisms	Acc: 93.6 Recall: 93.5 F1-Score: 93.9
	Density Estimation					Acc: 90.2 Recall: N/A F1-Score: N/A
[22]	Classification + Segmentation	Hard Sharing	Ultrasound	BUD (BUSI + BUSBRA + BUS UC + BUET_BUS)	ResNet18 Encoder, UNet decoder + Multi-Scale Fusion Module + Channel Attention Module	Acc: 87.5 Recall: 87.01 F1-Score: 87.54 Dice: 90.3
			Magnetic Resonance Imaging	BMD (BreaDM + Private Dataset)		Acc: 99.64 Recall: 99.71 F1-Score: 96.4 Dice: 91.5
[28]	Classification + Segmentation	Hard Sharing	Ultrasound	PRECISE BUS (BUSI + BrEaST + BUS-BRA)	ViT-B/16 backbone encoder + MGA Mechanisms	Acc: 90.7 Recall: N/A F1-Score: 88.7 Dice: 88.7

### 4.2. Federated Learning Methods for Breast Cancer Screening and Diagnosis

In the area of FL-based methods for BrC screening and diagnosis, most of the surveyed papers proposed simulated FL environments mostly using ultrasound images [29,30,31,32,33], mammograms [34,35,36,37,38,39,40,41], and histopathological images [42,43,44,45]. Table 4, Table 5, Table 6 and Table 7 synthetically present studies related to FL-based BrC screening and diagnosis.

First, most of the reviewed studies validated FL environments using multiple datasets. This practice not only strengthens their robustness, but also highlights their robustness. For instance, the study conducted by Elshenawy et al. [29] proposed an FL environment that was validated on three different datasets: Breast Ultrasound Images (BUSI) [14], Breast Ultrasound Lesion Segmentation Dataset (BUS-UCML) [46], and Breast Cancer Multi-modal Imaging Dataset (BCMID) [47]. For mammogram-based diagnosis, in addition to those utilized for MTL, the VINDR-MAMMO [48] and Chinese Mammography Database (CMMD) datasets [49] were employed. For histopathology-based diagnosis, BreakHis [30] was used because it contains data at multiple magnification levels.

Moreover, all FL environments used for BrC screening and diagnosis fall into the horizontal category. The authors considered the same imaging modality (i.e., ultrasound, mammography, and histopathological imaging) when constructing the FL environment. While some studies utilized a single dataset to create local clients [34,35,42,43,44,45], other studies simulated heterogeneous clients. In [29] a three-client FL system was employed using images belonging to different datasets for each client. Regarding the level of additional security introduced in the FL system, differential privacy was primarily used in the surveyed papers [33,39,41] as well as homomorphic encryption [36,45]. Although differential privacy is useful for increasing the security of FL environments by noise addition, it could decrease the performance of clients and increase overhead. Compared with MTL, fewer studies have utilized Grad-CAM diagrams to enhance the transparency of models [36,39,44].

Regarding the aggregation methods utilized, most of the reviewed papers employed FedAvg and FedProx. However, paper [50] utilized FedOpt as the aggregation technique. Compared to FedAvg and FedProx, FedOpt uses adaptive optimization on the server side while aggregating client-side weights. In conjunction with a ViT-based local model, the proposed FL environments showed superior performance (i.e., approximately 10–12% in terms of overall accuracy, recall and F1-Score) compared to the classical aggregation methods used in conjunction with CNN architectures. In addition, this study was conducted by Rehman. et al. [51] and incorporated a GAN-based data augmentation method at the client-side level rather than using it as a separate data pre-processing technique. Thus, the non-IID data were slightly attenuated by generating synthetic data prior to the aggregation phase.

**Table 4:** Summary on FL Methods for BrC Screening and Diagnosis on US images.

Ref.	Task Type	Aggregation Technique	Privacy Mechanism	Dataset	DL Architecture	Performance [%]
[29]	Multi-Class Classification	FedProx	Implicitly via federated architecture	BUSI	MobileNet	Acc: 80.92 Recall: N/A F1-Score: 78.13
					ResNet50	Acc: 81.57 Recall: N/A F1-Score: 78.58
					InceptionNetV3	Acc: 69.07 Recall: N/A F1-Score: 60.60
				BCMID	MobileNet	Acc: 61.29 Recall: N/A F1-Score: 54.00
					ResNet50	Acc: 53.62 Recall: N/A F1-Score: 45.29
					InceptionNetV3	Acc: 57.66 Recall: N/A F1-Score: 50.97
				BUS-UCLM	MobileNet	Acc: 77.71 Recall: N/A F1-Score: 69.81
					ResNet50	Acc: 72.00 Recall: N/A F1-Score: 65.62
					InceptionNetV3	Acc: 73.14 Recall: N/A F1-Score: 67.60
[31]	Segmentation	FedProx	Implicitly via federated architecture	BUSI + Dataset B	Attention-enhanced U-NET model	Acc: 96.07 Recall: 60.66 F1-Score: 70.76 Dice: 29.24
[32]	Segmentation	FedAvg	Implicitly via federated architecture	BUSI	Three-level encoder–decoder U-Net Model	Acc: 91.42 Recall: 24.09 F1-Score: 25.18
				Dataset B		Acc: 96 Recall: 21.37 F1-Score: 82.8
[33]	Classification and Segmentation	FedAvg	Differential Privacy with Gaussian Noise Injection	BUSI	Multi-Attention U-NET (Segmentation) ResNet50V2 + NASNetLarge + MAU-Net + meta-classifier (Classification)	Acc: 98.7 Recall: 91.11 F1-Score: 97.8 Dice: 89.72
				UDIAT		Acc: 96.82 Recall: 87.41 F1-Score: 97.8 Dice: 87.98
				BUSC		Acc: 96.92 Recall: 87.41 F1-Score: 90.32 Dice: 93.09
[50]	Classification	FedOpt	Client-side Differential Privacy	BUSI	ResNet50	Acc: 76.56 Recall: 76.56 F1-Score: 75.35
					VGG19	Acc: 85.05 Recall: 82.73 F1-Score: 83.05
					MobileNetV2	Acc: 66.44 Recall: 66.44 F1-Score: 59.15
					DenseNet121	Acc: 87.03 Recall: 87.03 F1-Score: 86.80
					ViT-small	Acc: 89.53 Recall: 89.53 F1-Score: 89.41
					CoAtNet	Acc: 88.51 Recall: 88.51 F1-Score: 88.46

**Table 5:** Summary on FL Methods for BrC Screening and Diagnosis on Mammograms.

Ref.	Task Type	Aggregation Technique	Privacy Mechanism	Dataset	DL Architecture	Performance [%]
[34]	Classification	FedAvg	Implicitly via federated architecture	DDSM	5–20-layer DNN	Acc: 89.7 Recall: 98.6 F1-Score: N/A
[35]	Classification	FedAvg	Implicitly via federated architecture	DDSM	DenseNet and Recurrent Neural Network	Acc: 95 Recall: 95.74 F1-Score: 95.76
[36]	Classification	FedAvg	Homomorphic Encryption	VINDR-MAMMO	3-layer Deep CNN	Acc: 97.1 Recall: 90.3 F1-Score: 93.71
				CMMD		Acc: 94.4 Recall: 92.3 F1-Score: 93.63
				INBreast		Acc: 91.6 Recall: 88.0 F1-Score: 90.43
[37]	Classification	FedAvg	Implicitly via federated architecture	3D Digital Breast Tomosynthesis	3-layer custom CNN architecture	Acc: 97.37 Recall: 96.88 F1-Score: N/A
[38]	Classification	FedAvg	Domain adversarial Training	Mammogram Dataset (KAUMDS)	ResNet	Acc: 98.8 Recall: 98.5 F1-Score: 98.2
[39]	Classification	FedAvg	Differential Privacy + Homomorphic Encryption	CBIS– DDSM	ResNet + EfficientNet with attention mechanisms	Acc: 93.7 Recall: N/A F1-Score: N/A
[40]	Segmentation	FedAvg	Implicitly via federated architecture	DDSM	VGG backbone feature extractor + UNet2 and Unet3	Acc: 91.4 Recall: 81.7 F1-Score: N/A Dice: 76.7
				CBIS-DDSM		Acc: 93.1 Recall: 78.9 F1-Score: N/A Dice: 75.2
				MIAS		Acc: 96.6 Recall: 99.3 F1-Score: N/A Dice: 86.9
				INBreast		Acc: 97.7 Recall: 98.0 F1-Score: N/A Dice: 76.4
[41]	Classification + Segmentation + Detection	FedAvg	Differential Privacy (in an ablation study)	INBreast	Pyramidal ViT + task-specific decoders	Acc: N/A Recall: N/A F1-Score: N/A Dice: 95.3

**Table 6:** Summary on FL Methods for BrC Screening and Diagnosis on Histopathological Images.

Ref.	Task Type	Aggregation Technique	Privacy Mechanism	Dataset	DL Architecture	Performance [%]
[42]	Classification	FedAvg	Homomorphic Encryption + Secure Multi-Party Computation + Differential Privacy	BreakHis	ResNet152	Acc: 84.39 Recall: N/A F1-Score: 67.45
					DenseNet201	Acc: 91.06 Recall: N/A F1-Score: 84.97
					MobileNetv2	Acc: 87.38 Recall: N/A F1-Score: 77.38
					EfficientNetB7	Acc: 84.02 Recall: N/A F1-Score: 72.78
[43]	Classification	FedAvg	Extended ElGamal Image Encryption	BreakHis	Custom CNN + twin attention modules	Acc: 95.68 Recall: 95.6 F1-Score: 95.63
[44]	Classification	FedAvg	Implicitly via federated architecture	BreakHis	Pretrained ResNet18 + self-attention modules	Acc: 95.95 Recall: 76.71 F1-Score: 77.68
[45]	Classification	FedAvg	Homomorphic Encryption	BreakHis	YOLOv6	Acc: 98 Recall: N/A F1-Score: N/A

Rather than utilizing direct medical images for BrC screening and diagnosis, several recent studies have utilized features extracted from digitized images of a fine needle aspirate (FNA) of a breast mass [51,52,53,54] using the Wisconsin Breast Cancer Dataset (WDBC) [55]. Due to the nature of the WDBC dataset, the studies presented in Table 7 mainly focused on classification tasks. Ref. [51] employed a custom GAN-based aggregation strategy to improve the performance and robustness of the FL-based WBCD classification model.

**Table 7:** Summary on FL Methods for BrC Screening and Diagnosis on features extracted from digitalized Images of FNA of breast masses.

Ref.	Task Type	Aggregation Technique	Privacy Mechanism	Dataset	DL Architecture	Performance [%]
[52]	Classification	FedAvg	Implicitly via federated architecture	WBCD	3-layer DNN	Acc: 97.5 Recall: 98.0 F1-Score: 97
[51]	Classification	Custom GAN-based aggregation	Differential Privacy	WBCD	Cramer GAN + custom 4-layer CNN architecture	Acc: 97.5 Recall: 96 F1-Score: 97
[53]	Classification	FedAvg	Differential Privacy	WBCD	2-layer Deep Neural Network	Acc: 96.1 Recall: 96.0 F1-Score: 97.0
[54]	Classification	FedAvg	Implicitly via federated architecture	WBCD	3-layer DNN with dropout layers	Acc: 98.25 Recall: 98.59 F1-Score: 98.59

### 4.3. Datasets Employed for Breast Cancer Analysis

Table 8 presents the datasets used for MTL- and FL-based BrC screening and diagnosis. First, from a clinical-based class distribution perspective, most datasets reflect a realistic split between medical images containing benign, malignant and healthy breast tissue (e.g., BUSI, UDIAT, OASBUD, BUS-UCML, INBreast or CBIS-DDSM). From a clinical perspective, the number of malignant observations is usually smaller than that of benign or healthy observations. Such an imbalance can bias the performance of DL models to the dominant class. Therefore, data augmentation techniques should be employed as pre-processing techniques to limit the bias risk and enhance the robustness of MTL models and FL environments. Second, almost all the datasets presented in Table 8, except for the CBIS-DDSM dataset, were collected from a single center. Therefore, to increase the generalizability of MTL and FL architectures, researchers should consider employing imaging data collected from multiple institutions during both training and inference stages. Third, the number of publicly available datasets corresponding to each imaging modality is different. While there are four main ultrasonography-based datasets, only three use mammography and histopathological imaging. Therefore, a future direction might be represented by the collection of datasets using mammography, whole-slide images, and histopathology.

**Table 8:** Datasets Used for Breast Cancer Screening and Diagnosis.

Dataset	Imaging Modality	Acquisition Center	Dataset Size	Class	Task
BUS [14]	Ultrasonography	Baheya Hospital, Cairo	600	Benign: 437	Classification, Segmentation
				Malignant: 210	
				Healthy: 133	
UDIAT [15]	Ultrasonography	UDIAT Diagnostic Centre, Parc Taulí University Hospital, Spain	163	Benign: 110	Classification, Segmentation
				Malignant: 53	
OASBUD [16]	Ultrasonography	Department of Ultrasound, Institute of Fundamental Technological Research, Poland	78	Benign: 52	Classification, Segmentation
				Malignant: 48	
BUS-UCML [46]	Ultrasonography	Ciudad Real General University Hospital	38	Benign: 174	Classification, Segmentation
				Malignant: 90	
				Healthy: 419	
CBIS-DDSM [17]	Mammography	Massachusetts General Hospital, Wake Forest University School of Medicine, Sacred Heart Hospital, and Washington University of St Louis School of Medicine	753 calcification cases 891 mass cases	Benign: 886	Classification, Detection
				Malignant: 758	
INBreast [18]	Mammography	Centro Hospitalar de São João, Portugal	115	Benign: 70	Classification, Detection
				Malignant: 45	
BreakHis [30]	Histopathology	P&D Laboratory, Brazil	82	Benign: 2480	Classification, Segmentation, Detection
				Malignant: 5429	
WDBC [55]	FNA Features	University of Wisconsin, USA	Not recorded	Benign: 357	Classification
				Malignant: 212	

## 5. Multi-Task and Federated Learning for Lung Cancer

### 5.1. Multi-Task Learning Methods for Lung Cancer Screening and Diagnosis

Research conducted on MTL- and FL-based LuC screening and diagnosis has mainly focused on computed tomography (CT) imaging, as shown in Table 9 and Table 10, mainly using the LIDC-IDRI [56], Chest CT-Scan [57], Lungs Disease Dataset 4 Types [58], and IQ-OTH/NCCD [59] datasets. Similar to the studies conducted on BrC screening and diagnosis, principal MTL-based approaches are represented by classification and segmentation [60,61,62,63]. However, several studies implemented hybrid [60] and soft [64] parameter sharing methods. Notably, the MTL models of most of the studies presented in Table 9 were not validated using external data. However, in [65], the authors utilized external data in addition to those used for training, to validate the model. For LuC screening and diagnosis, several studies have employed Grad-CAM diagrams as the XAI method, either in centralized MTL [62,66] or federated [67] configurations.

**Table 9:** Summary of MTL Methods for LuC Screening and Diagnosis.

Ref.	Tasks	Parameter Sharing	Imaging Technique	Dataset	DL Architecture	Performance [%]
[60]	Classification + Segmentation	Hybrid Sharing	CT	Lung PET CT Dx	StarNet-based encoder–decoder with edge uncertainty estimation	Acc: 88.4 Recall: 87.0 F1-Score: 84.6 Dice: 84.5
				STS		Acc: 86.7 Recall: 82.7 F1-Score: 83.0 Dice: 83.4
[68]	Detection	Hard Sharing	CT	LIDC-IDRI	YOLOv11 backbone with Feature Pyramid Network and Path Aggregation Network and anchor-based detection head	Acc: N/A Recall: 66.4 F1-Score: 76.3
	Multi-attribute regression					MAE: 51.6 RMSE: 71.9
[64]	Segmentation + PET	Soft Sharing	CT + PET Knowledge	NSCLC + Rad	Semi-supervised Student-Teacher Connected U-Net	Dice: 64.0 Recall: N/A
				NSCLC-Rad-Int		Dice: 38.0 Recall: N/A
				MSD Task06		Dice: 66.0 Recall: N/A
[69]	Classification + Image Reconstruction	Hard Sharing	CT	LUNA-16	Custom architecture of a 4-layer CNN	Acc: N/A Recall: 84.00 F1-Score: N/A
				LIDC-IDRI		Acc: N/A Recall: 87.74 F1-Score: N/A
[61]	Classification + Segmentation	Hard Sharing	CT	MedSeg	U-Net Convolutional Block Attention Module with MLP	Acc: 97.95 Recall: N/A F1-Score: N/A Dice: 89.81
				COVID-19 CT Lung and Infection Segmentation		Acc: 95.50 Recall: N/A F1-Score: N/A Dice: 89.03
				MosMedData: Chest CT Scans with COVID-19′		Acc: 97.27 Recall: N/A F1-Score: N/A Dice: 89.15
				COVID-19 CT segmentation		Acc: 98.14 Recall: N/A F1-Score: N/A Dice: 89.91
[62]	Classification + Segmentation	Hybrid Sharing	CT	LIDC-IDRI	Coarse and Segmentation Network	Acc: 91.9 Recall: 92.5 F1-Score: N/A Dice: 83.2
[70]	Histologic Subtype Classification	Hard Sharing	CT	Six combined datasets from The Cancer Imaging Archive (TCIA)	MobileNet MTL model with attention mechanisms	Acc: 91.4 Recall: 87.9 F1-Score: 93.61
	Clinical Staging Classification					Acc: 91.1 Recall: 89.3 F1-Score: 91.66
[63]	Classification + Segmentation	Hard Sharing	CT	LIDC-IDRI	U-Net + Classification Head	Acc: 72.92 Recall: N/A F1-Score: N/A Dice: 64.8
[66]	Adenocarcinoma Invasiveness Classification	Hard Sharing	CT	Private Dataset	Dense-Attention-based Knowledge Distilation Model	Acc: 98.6 Recall: 96.9 F1-Score: 97.1
	Tumor Growth Pattern Subtyping					Acc: 93.1 Recall: 93.9 F1-Score: 93.9
[65]	Adenocarcinoma and squamous cell carcinoma Classification	Hard Sharing	CT	TCIA: LUNG1 + Radiogenomics	ResNet block and Squeeze and Excitation Modules	Acc: 77.0 Recall: 81.2 F1-Score: N/A
[71]	Malignancy Classification	Hard Sharing	CT	Luna16	ResNet branch and 3D Swin ViT module	Acc: 92.61 Recall: 92.17 F1-Score: N/A
	Sphericity Classification					Acc: 91.63 Recall: 91.18 F1-Score: N/A
	Margin Classification					Acc: 92.12 Recall: 92.08 F1-Score: N/A
	Subtlety Classification					Acc: 91.63 Recall: 91.18 F1-Score: N/A

**Table 10:** Summary of FL Methods for LuC Screening and Diagnosis on CT Images.

Ref.	Task Type	Aggregation Technique	Privacy Mechanism	Dataset	DL Model Architecture	Performance [%]
[72]	Classification	Ensemble-based Aggregation	Implicitly via federated architecture	S0819 Lung Cancer	Deep Neural Network	Acc: 89.63 Recall: 81.26 F1-Score: N/A
[73]	Classification	FedAvg	Implicitly via federated architecture	Chest CT-Scan Dataset (Kaggle)	MobileNet	Acc: 90.61 Recall: 90.25 F1-Score: 90.75
					MobileNetv2	Acc: 92.27 Recall: 91.75 F1-Score: 92.25
					ResNet50v2	Acc: 88.95 Recall: 88.50 F1-Score: 89.25
					VGG16	Acc: 90.06 Recall: 90.50 F1-Score: 90.25
					Inceptionv3	Acc: 86.19 Recall: 87.00 F1-Score: 86.75
[74]	Detection	FedAvg	Implicitly via federated architecture	Luna16	3D VGG16 + Dual-path Faster R-CNN	Acc: 77.86 Recall: 77.54 F1-Score: 77.04
					3D ResNet18 + Dual-path Faster R-CNN	Acc: 83.41 Recall: 83.38 F1-Score: 83.40
					SumNet	Acc: 80.35 Recall: 80.0 F1-Score: 80.74
[75]	Classification	FedAvg	Implicitly via federated architecture	Chest CT-Scan Dataset (Kaggle)	KNN	Acc: 97.84 Recall: 98.1 F1-Score: 97.7
					Decision Tree	Acc: 96.04 Recall: 98.6 F1-Score: 97.5
					SVM	Acc: 95.87 Recall: 96.3 F1-Score: 96
[76]	Classification	FedAvg	Implicitly via federated architecture	IQ-OTH/NCCD LuC dataset	InceptionV3	Acc: 89.0 Recall: 80.0 F1-Score: 73.66
[77]	Segmentation	FedProx + Adaptive Weighted Aggregation	Implicitly via federated architecture	NSCLC-Radiogenomics	ViT encoder + Atrous spatial pyramid Pooling	Dice: 83.55 Recall: 90.15
				Medical Segmentation Decathlon (MSD)		Dice: 80.4 Recall: 90.98
[67]	Classification	FedAvg	Implicitly via federated architecture	Chest CT-Scan Dataset (Kaggle)	Custom CNN + Spatial and Channel Wise Attention Modules	Acc: 67 Recall: 67 F1-Score 65.8
[78]	Classification	FedAvg	Blockchain	Lungs Disease Dataset 4 Types (Kaggle)	DenseNet201	Acc: 90.0 Recall: 90.2 F1-Score: 89.8
[79]	Classification	FedAvg	Differential Privacy	IQ-OTH/NCCD Lung Cancer Dataset	ResNet101	Acc: 99.2 Recall: 98.7 F1-Score: 98.34
				Chest CT-Scan Lung Cancer Dataset		Acc: 98.7 Recall: 98.05 F1-Score: 97.52
[80]	Classification	FedAvg	Implicitly via federated architecture	IQ-OTH/NCCD Lung Cancer Dataset	ResNet50	Acc: 99.4 Recall: 99.03 F1-Score: 98.97
[81]	Classification	FedAvg	Implicitly via federated architecture	Lungs Disease Dataset 4 Types (Kaggle)	Custom 7-layer CNN	Acc: 89.96 Recall: N/A F1-Score: N/A
[82]	Segmentation	FedDus: Semi-supervised Aggregation	Implicitly via federated architecture	GDPH	U-Net	Dice: 93.48
				TJCH		Dice: 84.36
				CHSUMC		Dice: 83.28
				RIDER		Dice: 77.76
				INTEROBS		Dice: 88.70
				LUNG1		Dice: 84.60

### 5.2. Federated Learning Methods for Lung Cancer Screening and Diagnosis

The studies conducted on FL-based methods for LuC screening and diagnosis are presented in Table 10. Similar to studies conducted on BrC screening and diagnosis, the principal tasks were classified. However, most papers solely used federated architecture or differential privacy [79]. Gupta et al. [78] used blockchain technology to encrypt weights during both server-client and client-server updates.

Similar to the studies conducted on BrC screening and diagnosis, the primarily utilized aggregation techniques are represented by FedAvg and FedProx. However, several studies have utilized new and more efficient aggregation techniques [72,77,82]. This study was conducted by Abdelhamed. et al. [77] proposed a hybrid aggregation technique that combines FedProx with cosine similarity weighting. This approach addresses the non-IID problem from two perspectives: First, FedProx constrains the level of deviation of local updates during the training phase. Second, the adaptive aggregation at the server further downweights clients whose final updates diverge significantly from the global model. Therefore, compared with FedAvg, the hybrid aggregation technique increased the Dice coefficient by 0.75%. In [82], a semi-supervised FL environment that supported dynamic model aggregation was proposed. In contrast to FedAvg, which assigns aggregation weights based only on the size of the dataset and keeps them fixed during the training phase, the dynamic model aggregation method additionally incorporates local model quality, that is, the validation performance in the weighting process. In this way, if a local client with access to a large-scale dataset generalizes poorly, it would contribute less to the overall FL environment compared to FedAvg. Additionally, the study conducted by Sha et al. [67] analyzed the impact of the number of clients on the performance of the proposed FL environment. Therefore, a performance drop of approximately 26% between a three-client and five-client FL environment reflects increased data heterogeneity as the number of clients increases.

### 5.3. Datasets Employed for Lung Cancer Analysis

Table 11 presents the datasets used for the LuC analysis using the MLT and FL methods. The real-world clinical applicability varies across the reviewed datasets for LuC screening and diagnosis. The LIDC-IDRI dataset is, by far, the most comprehensive dataset, providing images collected from over one thousand patients. Similarly, multi-institutional datasets (e.g., LIDC-IDRI and IQ-OTH/NCCD) better approximate the real-world variability in scanner hardware and patient demographics. On the other hand, several datasets that are available via the Kaggle platform (e.g., Chest CT-Scan, Lungs Disease Dataset 4 Types) are richer in the provided classes (e.g., Adenocarcinoma, Squamous Cell Carcinoma, Tuberculosis). Therefore, in the context of FL-based LuC screening and diagnosis, different participating institutions can learn features extracted from multiple histological subtypes.

**Table 11:** Datasets Used for Lung Cancer Screening and Diagnosis.

Dataset	Imaging Modality	Acquisition Center	Dataset Size	Class	Task
LIDC-IDRI [56]	CT	Seven academic centers and eight medical imaging companies	1010	Nodule Non-nodule	Nodule detection
				Benign Malignant	Malignancy Classification
Chest CT-Scan [57]	CT	Not Explicitly Mentioned	Not Explicitly Mentioned	Adenocarcinoma: 338	Classification, Detection
				Large Cell Carcinoma: 187	
				Squamous Cell Carcinoma: 260	
				Healthy: 215	
Lungs Disease Dataset 4 Types [58]	CT	Not Explicitly Mentioned	Not Explicitly Mentioned	Bacterial Pneumonia: 2009	Classification, Detection
				Corona Virus: 2031	
				Tuberculosis: 2034	
				Viral Pneumonia: 2008	
				Healthy: 2013	
IQ-OTH/NCCD [59]	CT	Iraq-Oncology Teaching Hospital, National Center for Cancer Diseases	110	Benign: 120	Classification, Detection
				Malignant: 561	
				Healthy: 416	

## 6. Results and Discussions

### 6.1. Performance Comparison Achieved by Deep Learning and Federated Learning Models for Breast Cancer and Lung Cancer Screening and Diagnosis

**Figure 7:**
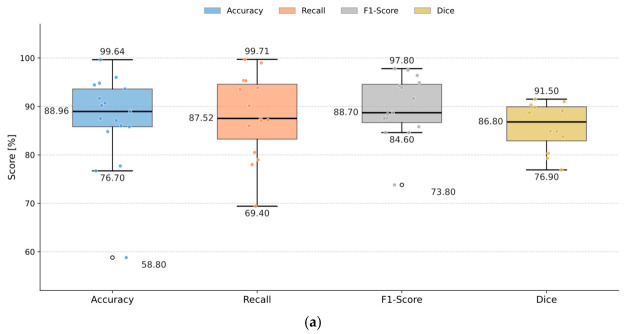

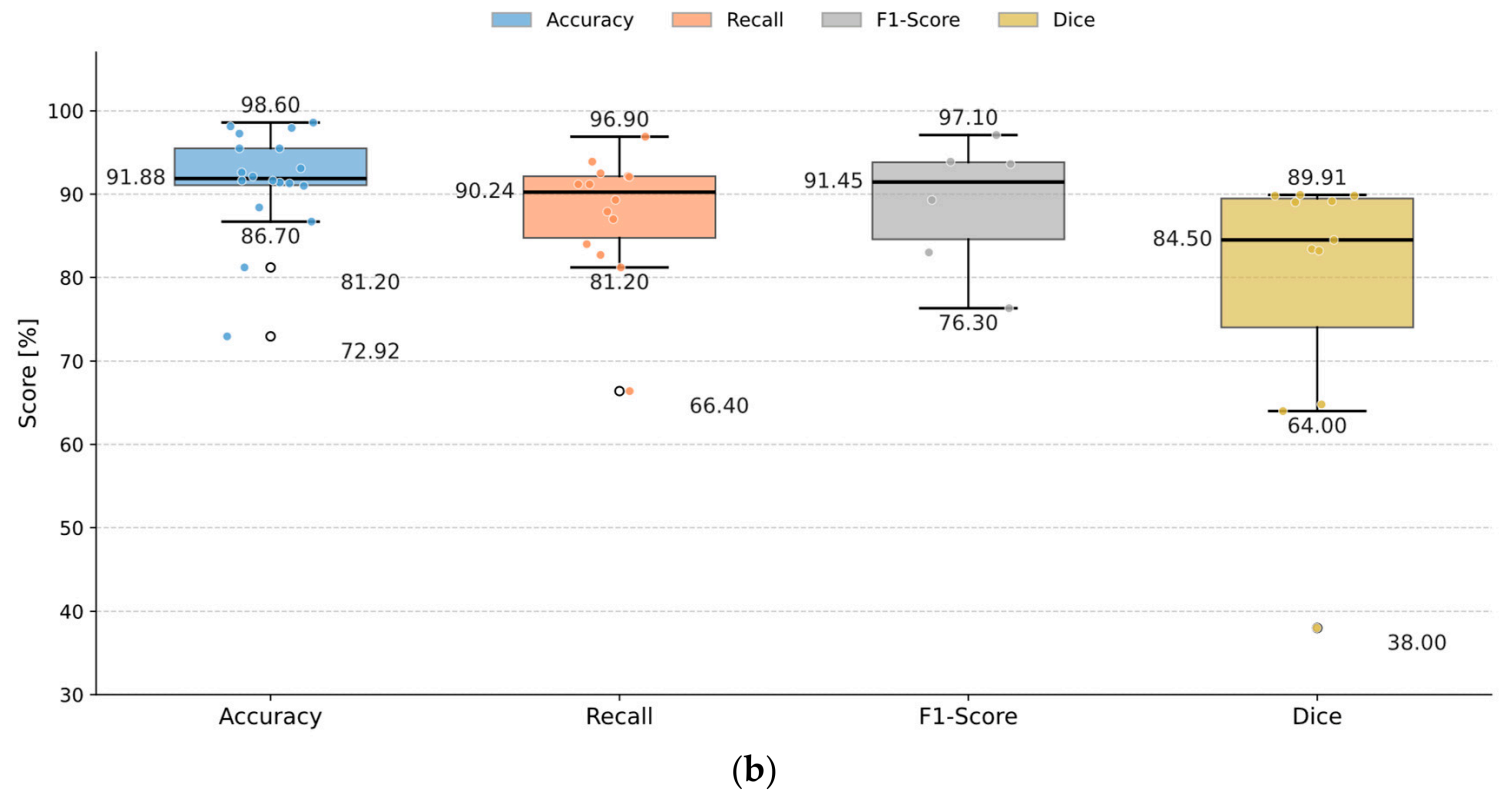
Box plots of the metrics obtained by the studies focused on MTL for (**a**) BrC and (**b**) LuC screening and diagnosis.

**Figure 8:**
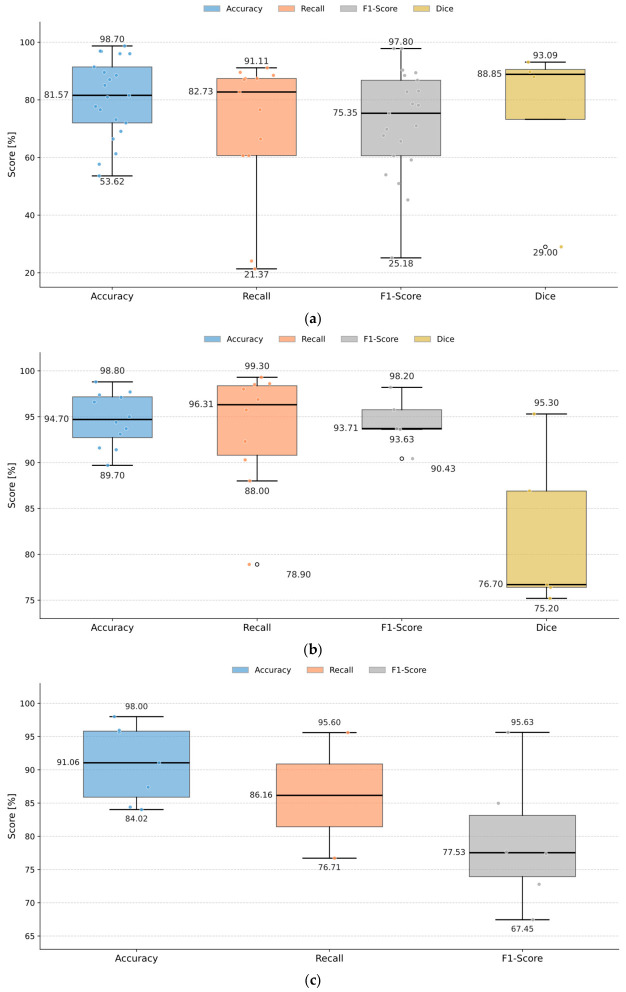

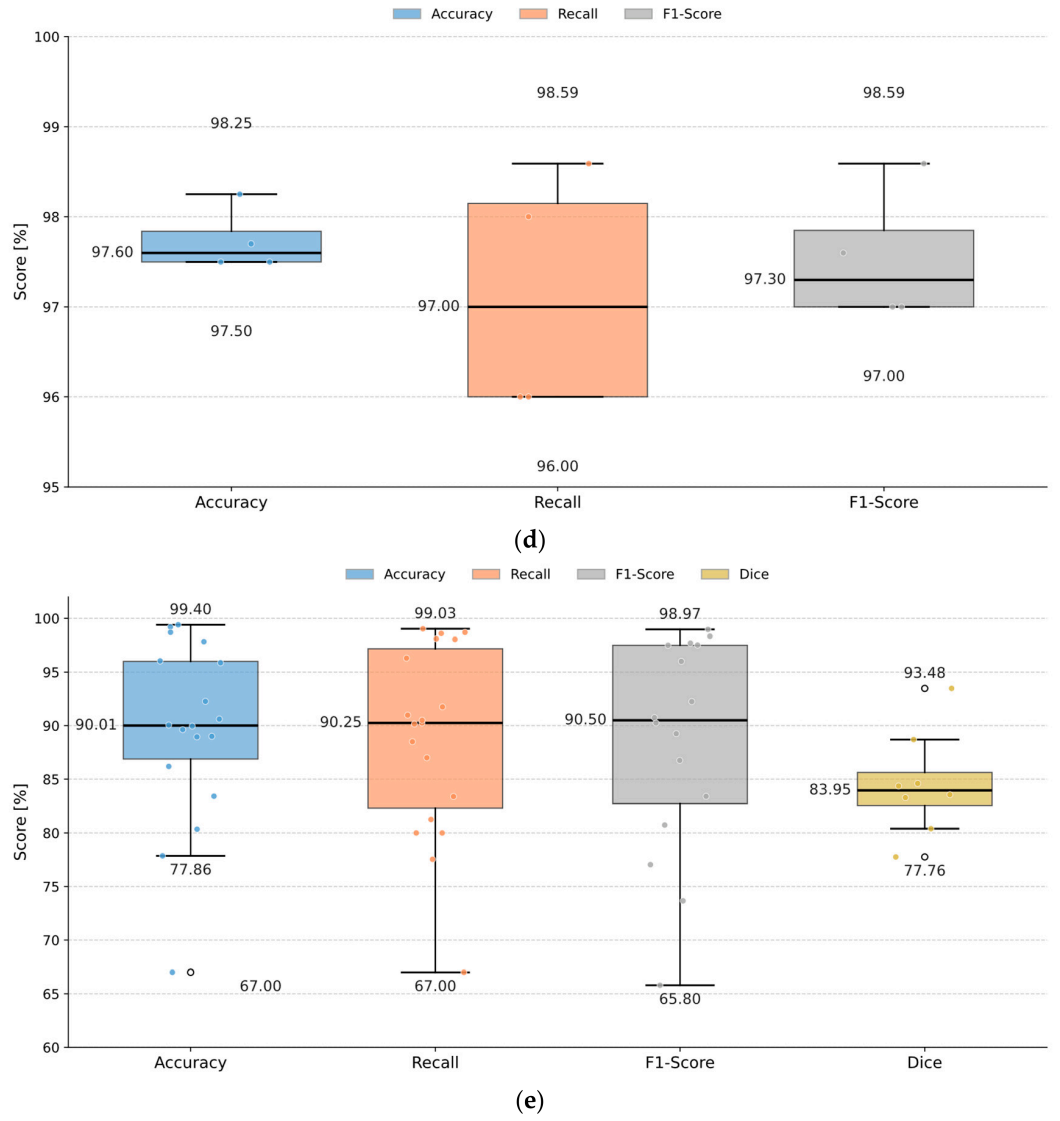
Box plots and jittered data points of the metrics reported by studies focused on FL for (**a**) BrC using ultrasound images, (**b**) BrC using mammograms, (**c**) BrC using histopathological images, (**d**) BrC using FNA data, and (**e**) LuC screening and diagnosis using CT images.

This subsection presents the results and discussion that can be drawn from previously reviewed studies. To obtain a better overview of the results obtained by the DL models in both the multitask and federated configurations, several box plots were created. Figure 7 and Figure 8 present box plots corresponding to the results of MTL- and FL-based screening and diagnosis for BrC and LuC, respectively.

The performance metrics utilized are represented by the accuracy (Equation (7)), recall (Equation (8)), F1-Score (Equation (9)), and Dice coefficient (Equation (10)).

(7) $$Accuracy=\frac{TP+TN}{TP+TN+FP+FN}$$

(8) $$Recall=\frac{TP}{TP+FN}$$

(9) $$F1-Score=2\cdot \frac{Precision\cdot Recall\;}{Precision+Recall}$$

(10) $$Dice\left(y,\;\hat{y}\right)=1-2\cdot \frac{\left|y\cap \hat{y}\right|\;}{\left|y\right|+\left|\hat{y}\right|\;}$$

where TP, TN, FP, and FN represent the numbers of true-positive, true-negative, false-positive, and false-negative predictions, respectively. In the case of the Dice coefficient, $\left|y\cap \hat{y}\right|$ represents tumoral area that was corectly predicted from the ground truth mask and $\left|y\right|+\;\left|\hat{y}\right|$ represents total number of pixels from both the ground truth and predicted mask. In addition to the classical accuracy metric, both the recall and F1-Score metrics were monitored. The recall metric is especially important in the context of medical image analysis because missing multiple true-positive values can significantly impact the diagnosis process by giving patients a false sense of safety. In addition, the F1-Score (i.e., the harmonic mean between the precision and recall) is particularly important when evaluating DL-based models on unbalanced datasets. The publicly available datasets used in the studies included in this survey were highly imbalanced, with the malignant class typically representing a minority class. Therefore, a high F1-Score implies that the model is not biased toward the majority class. Moreover, the Dice coefficient is also important for both BrC and LuC imaging analyses because it measures the overlap rate between the predicted and actual masks rather than the pixel accuracy. Thus, this metric is particularly reliable for irregular malignant tumors.

Figure 7a shows that the medians for accuracy, recall, and F1-score across the reviewed papers were relatively close. However, the recall was 1.44% smaller compared to the accuracy median value, highlighting that the MTL models tended to slightly miss the number of true positive values. Furthermore, the difference between the minimum values for accuracy and recall was larger (i.e., 7.3%), highlighting the sacrifice of recall in favor of the overall accuracy. The same pattern was observed for MTL-based LuC screening and diagnosis (Figure 7b). For both cancer types, the F1-Score median was relatively close to the median accuracy. However, the minimum values were also lower than those for recall and accuracy, further emphasizing the vulnerability of accuracy as a metric in imbalanced settings.

Regarding the performance obtained by the DL-based models integrated in the federated configuration for BrC screening and diagnosis (e.g., Figure 8a–d), the same pattern regarding the relationship between the accuracy, recall and F1-Score as in the case of the MTL cases is presented in Figure 7a. Additionally, compared with the centralized MTL approach, the median values for all considered metrics were similar. Therefore, the confidentiality of medical data can be preserved without compromising the performance of the DL-based models. Compared to the performance obtained by the studies focused on LuC screening and diagnosis (Figure 8d), the median values in terms of recall and F1-score median significantly outperformed ultrasound-based (Figure 8a), and histopathological-based (Figure 8c) methods.

### 6.2. Hardware and Software Used for Breast and Lung Cancer Screening and Diagnosis

In medical imaging analysis, particularly for BrC and LuC screening and diagnosis, the hardware equipment is a key factor that influences the training reliability of both DL models and FL environments. Since most of the utilized architectures in both centralized and federated configurations are represented by CNN and ViT models, the most employed piece of hardware that was used to train them is represented by the graphical processing unit (GPU). More specifically, Nvidia-based GPUs have been employed in the context of MTL for BrC [19,20,21,24,26,27,28] and LuC [60,64,68,69] screening and diagnosis as well as for FL methods on BrC [33,36,37,38,39,40] and LuC [74,77] screening and diagnosis. The main advantage of Nvidia GPUs in the DL-based analysis of medical imaging is represented by CUDA, which enables the capability to run thousands of parallel threads simultaneously on GPU cores, thereby accelerating matrix computations. Most of the reviewed papers used high-end Nvidia GPUs from the RTX [19,20,26,27,33,38,39] and GTX families [21,24,36,40]. Both GPU families are highly reliable for DL-based medical-image analyses. However, GPUs belonging to the RTX family provide support for tensor cores, which accelerates training and improves memory management [83]. Additionally, several studies utilized the Google Colab platform, which exposes the Nvidia T4 GPU [37,74,77] for free for a limited period of time. This platform is also reliable for users with low-end hardware, who want to use the power offered remotely by the T4 GPU in their browser.

The software frameworks that are employed to train and validate DL and FL models for BrC and LuC screening and diagnosis are mainly represented by PyTorch (versions 1.11-2.1) [19,20,21,40], and TensorFlow (version 1.13-2.21) and/or Keras [24,27,33,37,38]. From the model training point of view, PyTorch [84] is more verbose and user-friendly because it offers the capability of implementing custom training and validation loops, which can significantly improve the development process. On the other hand, Tensorflow [85] is more focused on fast model and architecture development, offering out-of-the-box methods for implementing the training and validation pipelines (i.e., the fit method).

### 6.3. Integration of Multi-Task Learning Models Within Federated Learning Environments

Currently, most DL-based models for BrC and LuC screening and diagnosis fall into two principal categories. The first category is represented by centralized MTL-based architectures that can simultaneously predict two different tasks at the same time using centralized medical-imaging data. The second category comprises unimodal architectures (i.e., mostly focused on classification and segmentation) that are integrated within horizontal FL environments.

An emerging topic is the integration of MTL models into FL environments. Currently, several studies have investigated this combination, specifically for BrC screening and diagnosis [33,41]. The paper conducted by Raheem. et al. [33] proposed a dual-stage MTL-based model for the segmentation and classification of breast lesions. While the segmentation branch was represented by an attention-enhanced U-Net model, the classification branch was represented by an ensemble model composed of ResNet50V2, NASNetLarge, and MAU-Net feature extractors. In addition, the paper conducted by Nath. et al. [41] proposed a ViT-based model that is able to learn task-agnostic features using a shared encoder and employ task-specific decoders for reliable feature extraction. The proposed MTL-based model trained and validated in a federated configuration achieves competitive performance on INBreast (i.e., achieving 95.3% in terms of the Dice coefficient).

Therefore, several challenges have been identified. First, there is a significant lack of professionally annotated datasets containing both medical images and corresponding masks provided by experienced radiologists [33]. Therefore, utilizing MTL-based models in federated configurations can leverage partially labeled imaging data that are common in clinical settings by distributing task-specific supervision across the entire FL environment. Another major challenge when employing MTL-based models in federated configurations is related to the vulnerability of the model’s parameters during knowledge transfer between the clients and the server [33], as they are very sensitive to various external attacks, such as Byzantine attacks and weight poisoning inference attacks. Another challenge in this context is inconsistent data distributions and varying imaging modalities as factors that introduce catastrophic forgetting, noisy optimization, and instability in model convergence [86]. However, several advantages of integrating MTL architectures into FL environments can be outlined. First, the amount of time and resources can be significantly reduced by utilizing semi-supervised MTL models within FL environments due to their capability to handle partially labeled images. Second, training across heterogeneous medical institutions exposes both global and local models to increased variability in terms of medical imaging, reduces overfitting to the distribution of a single model, and improves the real-world reliability. Third, MTL-based modes can reduce communication costs compared to the utilization of separate unimodal architectures at the client-side level. Therefore, this aspect is especially important in bandwidth-constrained environments such as rural hospitals.

### 6.4. Challenges and Future Directions

Based on the reviewed papers, several challenges might arise for MTL- and FL- screening and the diagnosis of BrC and LuC. First, data heterogeneity is a critical challenge. For MTL-based diagnosis, most reviewed papers employ rigid state-of-the-art feature backbone extractors, such as InceptionNet, MobileNet, or ResNet, and utilize specialized heads per task. Therefore, rather than employing such rigid feature extractors, lightweight adaptive layers can be used for every task to increase the reliability and efficiency of the feature extraction process.

Regarding the existing benchmarking and evaluation protocols, it is observed in Table 3, Table 4, Table 5, Table 6, Table 7, Table 8 and Table 9 that the reviewed papers do not consider a standard set of performance metrics. In fact, the metric that was mostly utilized was accuracy, while recall and F1-Score were excluded. Therefore, to ensure a consistent benchmarking system, the accuracy, precision, recall and F1-Score should be utilized in conjunction with classification tasks, while the Dice coefficient and intersection over union should be utilized for segmentation tasks.

External robustness refers to the capability of DL-based models to perform well on datasets that are not used during internal training. Additionally, this validation is especially important in centralized configurations. Even though external validation is critical in assessing the reliability of DL-based architectures, most of the MTL models are solely validated using internal parts of the datasets used for training. While some studies conducted on centralized MTL-based BrC screening and diagnosis admit this limitation [27], several studies conducted on centralized LuC screening and diagnosis validate their models on external cohorts [64,65]. Usually, external validations diminish the performance of the DL models due to difference in data distribution. In fact, paper [65] highlights this performance drop from 0.843 AUC obtained on the internal dataset to 0.732 AUC obtained on the external dataset.

For FL-based screening and diagnosis systems, most of the reviewed studies implemented horizontal federated systems, in which the clients were assumed to share the same data distribution, both for BrC screening and diagnosis [34,35,38,43,51,52,53,54], and for LuC screening and diagnosis [67,72,73,76,80,81]. This assumption may limit their practical applicability, since in real-world scenarios, each client may contain data from different distributions. Additionally, an important research direction in FL concerns the analysis of server-side and client-side component roles. The predominant focus on overall model performance often limits the detailed investigation of the distinct effects of local training strategies, client heterogeneity, and server aggregation mechanisms [32,80]. However, only a few studies have clearly distinguished between the performance of the global server and that of individual clients [53]. Nonetheless, most FL systems for BrC and LuC screening and diagnosis focus on a single task, predominantly classification.

Taking into account the aforementioned limitations, we propose the following future directions:

- Development of hybrid MTL models using a mixed approach composed of both hard and soft parameter sharing.
- Development of standardized benchmark systems that incorporate recall and F1-Score to better reflect the performance of DL-based models.
- Development of Vertical and Transfer learning FL systems in the context of BrC and LuC screening and diagnosis.
- Development of FL systems with clients containing data from different distributions.
- Development of FL systems trained and validated on edge devices (i.e., Raspberry Pi or Nvidia Jetson Nano).
- Utilization of blockchain technology as additional security method for FL environments.
- Development of FL systems incorporating MTL-based models for BrC and LuC screening and diagnosis [33,41].
- The development of multi-modal Vision Language Models (VLM) in both centralized and federated configurations can fuse visual information extracted from medical images with language knowledge to improve the understanding of medical data and clinical decision-making processes [87].
- Development of knowledge distillation-based systems for BrC and LuC screening and diagnosis [88].
- Future reviews could include an analysis of unimodal-based methods for BrC and LuC screening and diagnosis to compare their performance with that obtained by the MTL- and FL-based techniques to determine which method is more feasible from a clinical viewpoint.
- To monitor the consistency of clients with the global server, we recommend using the relative deviation metric as shown in Equation (11).

(11) $${RelativeDeviation}_{client}^{M}=\frac{M_{client}-M_{server}}{M_{server}}$$

where M represents a performance metric (i.e., accuracy, recall, Dice coefficient, or F1-Score) recorded at either the client or the server level. If the relative deviation is negative, the predictions of the local models are more consistent than those of the global model and vice versa. Thus, a coherent comparison can be performed between the reliability of each client relative to the global model. Additionally, the main advantage of this approach lies in its metric- and data-agnostic nature, which allows it to be applied to FL environments utilizing any type of medical imaging technology, thereby ensuring consistency measurement across any performance metric.

## 7. Conclusions

BrC and LuC are two aggressive types of cancer that affect both men and women worldwide. Additionally, medical data are usually difficult to collect because of various regulations imposed by medical institutions. This paper presents a survey of MTL and FL methods for BrC and LuC screening and diagnosis. Based on the surveyed papers, the FL-based models achieved comparable performance to the MTL-based model, thus highlighting their efficiency in preserving data confidentiality without compromising the performance. The main challenges for both MTL- and FL-based methods are represented by heterogeneous imaging data as well as a lack of standardization in benchmarking protocols.

Future directions can be represented on one hand by the development of FL environments containing hybrid parameter-sharing MTL models, personalized aggregation techniques that address data heterogeneity, knowledge distribution-based architectures [88], and edge-based FL models. However, future studies might develop centralized and decentralized VLM-based architectures that fuse both imaging and language information to increase the reliability of BrC and LuC screening and diagnosis processes [87].

## Data Availability

No new data were created or analyzed in this study.

## Abbreviations

The following abbreviations are used in this manuscript:

BrC	Breast Cancer
CAD	Computer-Aided Diagnosis
CNN	Convolutional Neural Networks
CT	Computed Tomography
DL	Deep Learning
DNN	Deep Neural Network
FL	Federated Learning
FNA	Fine Needle Aspirate
FP	False Positive
FN	False Negative
GAN	Generative Adversarial Network
GPU	Graphics Processing Unit
KNN	k-nearest Neighbors
LuC	Lung Cancer
MTL	Multi-Task Learning
PET	Positron Emission Tomography
PRISMA	Preferred Reporting Items for Systematic Reviews and Meta-Analyses
SVM	Support Vector Machines
TN	True Negative
TP	True Positive
ViT	Vision Transformer
